# Ovalbumin-containing core-shell implants suitable to obtain a delayed IgG1 antibody response in support of a biphasic pulsatile release profile in mice

**DOI:** 10.1371/journal.pone.0202961

**Published:** 2018-08-30

**Authors:** Katie Amssoms, Philip A. Born, Max Beugeling, Ben De Clerck, Ellen Van Gulck, Wouter L. J. Hinrichs, Henderik W. Frijlink, Niels Grasmeijer, Guenter Kraus, Roger Sutmuller, Kenny Simmen, Lieven Baert

**Affiliations:** 1 Discovery Sciences, Janssen Research & Development, a division of Janssen Pharmaceutica NV, Beerse, Belgium; 2 Department of Pharmaceutical Technology and Biopharmacy, University of Groningen, Groningen, The Netherlands; 3 Infectious Diseases & Vaccines Therapeutic Area, Janssen Research & Development, a division of Janssen Pharmaceutica NV, Beerse, Belgium; 4 Johnson & Johnson Innovation Center, London, United Kingdom; 5 Jalima Pharma bvba, Brugge, Belgium; Chapman University, UNITED STATES

## Abstract

A single-injection vaccine formulation that provides for both a prime and a boost immunization would have various advantages over a multiple-injection regime. For such a vaccine formulation, it is essential that the booster dose is released after a certain, preferably adjustable, lag time. In this study we investigated whether a core-shell based implant, containing ovalbumin as core material and poly(DL-lactic-*co*-glycolic acid) of various monomer ratios as shell material can be used to obtain such a booster release. An *in vitro* release study showed that the lag time after which the ovalbumin was released from the core-shell implant increased with increasing lactic to glycolic acid ratio of the polymer and ranged from 3–6 weeks. Fluorescence spectroscopy showed minimal differences between native ovalbumin and ovalbumin from core-shell implants that were incubated until just before the observed *in vitro* release. In addition, mice immunized with a subcutaneous inserted core-shell implant containing ovalbumin showed an ovalbumin-specific IgG1 antibody response after a lag time of 4 or 6–8 weeks. Moreover, delayed release of ovalbumin caused higher IgG1 antibody titers than conventional subcutaneous vaccination with ovalbumin dissolved in PBS. Collectively, these findings could contribute to the further development of a single-injection vaccine, making multiple injections of the vaccine superfluous.

## Introduction

Vaccination is one of the most effective and efficient ways to control the spread of more than thirty infectious diseases. Worldwide, approximately 2–3 million deaths every year are prevented by the use of vaccines [1]. Currently, most frequently used vaccines are subunit vaccines consisting of peptides and proteins or are based on polysaccharides [2,3]. After vaccination, the adaptive immune system learns from the vaccine to create immunological memory in order to provide protective immunity against future pathogen encounters. Because subunit-based vaccines and polysaccharide-based vaccines are often not highly immunogenic, usually a second or even a third administration of the vaccine (booster) after an earlier administration (primer) is required to acquire sufficient efficacy. Usually, vaccines are administered by injection, and thus the multiple-injection regime has the disadvantage that it jeopardizes the compliance of the vaccinee. Another disadvantage is that multiple-injection regimes are difficult to employ in remote areas of developing countries [4].

A possible solution would be the development of a vaccine formulation that provides for a prime-boost immunization after a single-injection, instead of a multiple-injection regime. Over the past 40 years, there have been several attempts to develop such vaccine formulations. Most of the work done was focused on monolithic systems consisting of a biodegradable polymer in which the antigen is incorporated [5]. The problem of most of these systems is that as biodegradation continuously proceeds, the antigen is also continuously released, which might induce tolerance towards the antigen rendering the vaccination ineffective [6].

An ideal injectable single-administration vaccine should consist of a controlled release implant from which the antigen is partially immediately released (primer), while the remaining part is released after a certain lag time (booster), depending on the type of vaccine. We hypothesize that biocompatible and biodegradable polymers might be applicable in such a pulsatile delivery system and serve as a barrier, providing the lag time necessary for the booster immunization. Such a barrier should be non-swellable and nonporous to avoid continuous release of the antigen by diffusion. We envisage that poly(DL-lactic-*co*-glycolic acid) (PL(G)A) might serve as a barrier layer or shell surrounding a core containing the antigen. A certain period of time after administration, the polymer has degraded to such an extent that it cannot act as a barrier anymore, which subsequently leads to a delayed release of the antigen [7]. Since the lactic to glycolic acid ratio of PLGA highly influences its degradation rate [8,9], we envisage that the lag time prior to the boost release can be tailored by changing the monomer ratio.

In the study described in this paper, we investigated the feasibility of a single-administration vaccine by using a core-shell based implant. The focus in the present study was on obtaining the boost release after a certain lag time, since this is the most challenging aspect of such a single-administration vaccine. To investigate whether the release of a model antigen after a certain lag time is possible, an *in vitro* experiment was conducted where the lactic to glycolic acid ratio of a PLGA shell, surrounding an ovalbumin (OVA) containing core, was varied. Intrinsic and extrinsic fluorescence spectroscopy was performed to investigate whether OVA maintained its native conformation within the core during the lag time. Based on the results, an *in vivo* experiment in mice was conducted. A core-shell implant containing the model antigen, OVA, was surgically inserted subcutaneously (s.c.) to determine whether antibody titers were indeed induced after a certain lag time. We also investigated whether a biphasic induction of OVA-specific antibodies was possible by priming mice with a s.c. injection of an OVA solution at the same time of the surgical insertion of the core-shell implant.

## Materials and methods

### Materials

Lyophilized OVA powder (protein ≥ 98%; 44.3 kDa), sodium carbonate, sodium tartrate dibasic dihydrate, copper(II)sulfate pentahydrate, sodium deoxycholate, trichloroacetic acid, 8-anilino-1-naphthalenesulfonic acid ammonium salt (ANS), bovine serum albumin (BSA), Tween 20, and a monoclonal anti-OVA-IgG1 antibody were from Sigma Chemical Co. (St. Louis, MO, United States). Inulin (4 kDa) was a generous gift from Sensus (Roosendaal, The Netherlands). Poly(DL-lactic-*co*-glycolic acid) (PLGA) with lactic to glycolic acid ratios of 50:50 (PURASORB^®^PDLG 5002), 75:25 (PURASORB^®^PDLG 7502), and poly(DL-lactic acid) (PLA, PURASORB^®^PDL 02), all with an intrinsic viscosity of 0.2 dL/g and a molecular weight of 17 kDa, were a gift from Corbion Purac Biomaterials (Gorinchem, The Netherlands). Sodium dihydrogen phosphate dihydrate, disodium hydrogen phosphate dihydrate, potassium dihydrogen phosphate, sodium hydroxide, sodium dodecyl sulfate, and Folin-Ciocalteu’s phenol reagent were from Merck KGaA (Darmstadt, Germany). Sodium chloride and mannitol were from BUFA (IJsselstein, The Netherlands). Sodium azide was from Acros Organics (Geel, Belgium). Blood collection tubes were from Sarstedt AG & Co (Nümbrecht, Germany). Flat-bottomed 96-well Nunc-Immuno plates with MaxiSorp surface and phosphate-buffered saline (PBS) were from Thermo Fisher Scientific (Waltham, MA, United States). Biotin-conjugated goat anti-mouse IgG1 and streptavidin-horseradish peroxidase (HRP) conjugate were from Southern Biotech (Birmingham, AL, United States). 3,3’,5,5’-tetramethylbenzidine (TMB) was purchased from Amresco LLC (Solon, OH, United States). Sulfuric acid 1 M/2 N was from VWR International Ltd (Leicestershire, England). All solvents were reagent grade and from VWR International Ltd (Leicestershire, England).

### Production of core-shell implants: Production of oblong cores

The core containing OVA stabilized with inulin was prepared as follows. An amount of inulin (1.25 g) was dissolved in 25 mL of 5 mM phosphate buffer solution (pH 7.0) under continuous heating on a hot plate (80 °C) in a closed container. After the inulin was completely dissolved, the solution was allowed to cool down to room temperature (20 °C). OVA (100 mg) was gently dissolved in 20 mL of this solution, resulting in a solution with an OVA:inulin weight ratio of 1:10. Injection vials (10 mL) were charged with 2 mL of the solution. The solutions were rapidly frozen by immersing the vials in liquid nitrogen and then immediately placed on the shelf of a freeze-dryer (Christ Epsilon 2–4 LSC, Salm & Kipp, Breukelen, The Netherlands), pre-cooled at -35 °C. Primary drying was performed for 32 h at a pressure of 0.220 mBar. Secondary drying was done for 12 h at a pressure of 0.055 mBar, while the temperature was gradually increased to 20 °C. The obtained powder was stored under dry nitrogen gas and the vials were hermetically sealed.

The core (oblong: 6 x 2 x 2 mm, ~ 25 mg) consisted of a physical mixture of OVA freeze-dried with inulin in a 1:10 w/w ratio (44 wt-%) and mannitol (56 wt-%), and was produced using an ESH compaction apparatus (Hydro Mooi, Appingedam, The Netherlands). A compaction load of 3 kN, a compaction rate of 0.5 kN/s, and a hold time of 0.1 s was used. The compaction of the physical mixture resulted in a core with a disintegration time of ≤ 120 s and a crushing strength of ≥ 5 N. Placebo cores composed of 44 wt-% freeze-dried inulin and 56 wt-% mannitol were made in exactly the same way.

### Production of core-shell implants: Compression coating of oblong cores with PLGA or PLA

**Fig 1:**
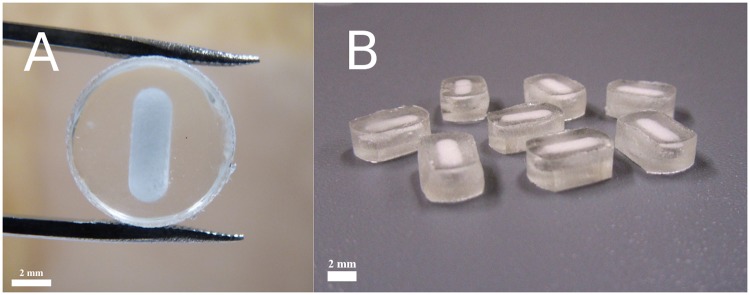
A-B. Top view of a core-shell implant for the *in vitro* release study (A). Core-shell implants for the *in vivo* study (B). Oblong cores of 25 mg containing 44 wt-% freeze-dried OVA with inulin (1:10 w/w ratio) and 56 wt-% mannitol were compression-coated with 250 mg of PL(G)A. The final shape (≈ 5 x 9 mm) of the core-shell implants for the *in vivo* study was obtained by abrasion.

A technique similar to that described by Guse et al. was used for the compression coating step [10]. In short, PLGA with different lactic to glycolic acid ratios (50:50 and 75:25) or PLA (PLGA 100:0) as received, was first ground for 5 s in a grinder (Moulinex AR100G31, Écully, France) to reduce the particle size of the polymer. To produce a PLGA or PLA layer around the oblong cores, first 125 mg was compressed using a preheated (48 °C) tablet die (9 mm) with a compaction load of 5 kN, a compaction rate of 0.5 kN/s, and a hold time of 10 s. The oblong core containing OVA as active compound was then directly placed in the middle of the PLGA- or PLA-containing die, followed by the filling with an additional layer of 125 mg PLGA or PLA, and compressed using the same settings. Before removing the compression-coated core from the die, the die was heated for 20 min at 48 °C. After this heating step, the PLGA- or PLA-coated core was re-compressed using a compaction load of 0.5 kN, a compaction rate of 0.5 kN/s, and a hold time of 10 s to close any pores. A number of core-shell implants were compressed under vacuum to remove air from the polymer layer. Fig 1A and 1B show the resulting core-shell implants for the *in vitro* release study and *in vivo* study, respectively. Its transparent appearance indicates that the shell was nonporous.

### *In vitro* release study: The delayed release of OVA and the influence of lactic to glycolic acid ratio on lag time

To investigate the influence of lactic to glycolic acid ratio on lag time, different types of PLGA and PLA were used, i.e. lactic:glycolic acid in a ratio of 50:50, 75:25, and PLA consisting of 100% poly(DL-lactide) (PLGA 100:0). Core-shell implants containing 1 mg OVA were placed in closable injection bottles (one per bottle, n = 6) containing 10 mL of 100 mM PBS (pH 7.4) as release buffer and placed in a shaking water bath (80 rpm) at 37 °C. Sodium azide (0.02% (w/v)) was added to the release buffer to prevent bacterial growth.

Samples of 1.2 mL were taken at predetermined time points and 1.2 mL of fresh preheated (37 °C) release buffer was pipetted back into the injection bottle to keep the volume constant. The samples were first centrifuged at 13,000 rpm for 5 minutes to remove any particulates (degradation products of the polymer) that could interfere with the measurement. An amount of 1 mL of the supernatant was then transferred into a new tube on which a modified Lowry protein assay as described by Tonnis et al. was conducted to determine the protein content [11].

### Fluorescence spectroscopy and OVA conformation

A fluorescence spectroscopic method similar to that described by Lechevalier et al. [12] was used to investigate whether OVA maintained its native conformation directly after freeze-drying (OVA-FD) and subsequent storage for one week at 60 °C as freeze-dried powder (OVA-FD-60 °C). In addition, the conformation of OVA within the core during the lag time was studied. To investigate this, OVA-PLGA 50:50 and OVA-PLGA 100:0 core-shell implants (n = 3 for each polymer) were incubated as previously described for the *in vitro* release study. The OVA-PLGA 50:50 and OVA-PLGA 100:0 core-shell implants were incubated for 21 and 36 days, respectively, just before OVA release was observed during the *in vitro* release study.

After incubation, the release medium was removed and the core-shell implants were cut to pieces using a scalpel. An amount of 20 mL 1X PBS was added to the core-shell pieces to dissolve the OVA core. The OVA containing solution was then filtered through a 1 μm syringe filter (GE Healthcare, Chicago, IL, United States) to obtain the final sample. An unprocessed native OVA solution 1X PBS (native OVA) and an OVA solution 1X PBS exposed to a heat shock for 30 minutes at 100 °C (HS-OVA) were used as positive and negative control, respectively. The previously described Lowry protein assay was performed to determine the protein concentration, which was adjusted if necessary. For all fluorescence measurements, placebo core-shell implants and 1X PBS were used to correct for background signal of the OVA containing core-shell implants and the native OVA solutions, respectively. Each sample was measured in triplicate.

#### Intrinsic fluorescence spectroscopy

To measure the intrinsic fluorescence, a fluorescence Quartz cuvette (L = 10 mm, Hellma GmbH & Co. KG, Müllheim, Germany) was filled with 1.5 mL sample. The cuvette was placed in the sample holder and the sample was gently stirred during measurement to prevent photobleaching. The fluorescence of the samples was measured by a fluorospectrometer (Quantamaster 40, Photon Technology International, Inc., Birmingham, NJ, United States) using an excitation wavelength of 295 nm, a slit width of 2.5 nm, and a temperature of 20 °C. The emission of the sample was recorded from 300 to 360 nm.

#### Extrinsic fluorescence spectroscopy

Directly after measuring the intrinsic fluorescence of the sample, 18.8 μL of a 2 mM ANS solution in 1X PBS was added to the 1.5 mL sample containing cuvette to measure the extrinsic fluorescence. For extrinsic fluorescence spectroscopy, an excitation wavelength of 386 nm was used and the emission of the sample was recorded from 400 to 600 nm.

### Animals

Eight- to twelve-week-old female BALB/c mice, 20–25 g, were obtained from specific pathogen-free facilities at Janvier (Le Genest-St-Isle, France). The mice were maintained and handled in our animal facilities in accordance with the institutional and national guidelines. All experiments were conducted under approval from the Janssen Ethics Committee on Animal Experiments. The permit number obtained from the Janssen Ethics Committee on Animal Experiments was ‘Proj 019 OVA’.

### Mouse immunization and sample collection

Thirty-nine mice were divided into 8 groups: the treatment groups (groups A and B) contained 6 mice. The three positive control groups (groups C, D, and E) contained 6, 3, and 6 mice, respectively. The placebo groups (groups F, G, and H) contained 3, 3, and 6 mice, respectively. Table 1 gives an overview of the groups used for the immunization experiment. Mice of group A were immunized with the OVA-containing PLGA 50:50 core-shell implant, while group B was immunized with the OVA-containing PLGA 100:0 core-shell implant. The mice of groups C and D were immunized s.c. with a prime vaccination of OVA (1 mg) in 1X PBS solution (200 μL, pH 7.2), together with an OVA-containing PLGA 50:50 and PLGA 100:0 core-shell implant, respectively. The mice of group E were immunized s.c. with OVA (1 mg) in 1X PBS solution (200 μL). A booster immunization of OVA (1 mg) in 1X PBS (200 μL) was given 21 days later. Group F was immunized with the placebo-PLGA 50:50 core-shell implant, while group G was immunized with the placebo-PLGA 100:0 core-shell implant. To investigate the potential adjuvant effect of PLGA, group H was immunized with the placebo-PLGA 50:50 core-shell implant, an immunization of OVA (1 mg) in 1X PBS (200 μL) was given 21 days later.

**Table 1:** Overview of the formulation composition used per group of mice immunized subcutaneously.

Group	Formulation composition	Amount OVA
A	OVA-PLGA 50:50 core-shell implant	1 mg
B	OVA-PLGA 100:0 core-shell implant	1 mg
C	OVA-PBS + OVA-PLGA 50:50 core-shell implant	1 mg + 1 mg
D	OVA-PBS + OVA-PLGA 100:0 core-shell implant	1 mg + 1 mg
E	OVA-PBS liquid injection (prime-boost)	1 mg + 1 mg
F	Placebo-PLGA 50:50 core-shell implant	-
G	Placebo-PLGA 100:0 core-shell implant	-
H	Placebo-PLGA 50:50 core-shell implant + OVA-PBS	1 mg

The mice of groups A-D and F-H received the core-shell implant using a surgical procedure. Briefly, 15 minutes before actual surgery, mice were treated with the analgesic Meloxicam (Metacam^®^, Boehringer Ingelheim, Germany, 5 mg/kg, s.c.). Following isoflurane anesthesia, a small incision through the skin of the right abdominal side was made. Sterile forceps and blunt end scissors were used to separate the skin and to create a subcutaneous pocket, in which the core-shell implants were placed on top of the s.c. musculature. The skin was then apposed and stapled with surgical clips. After the procedure, mice were moved to a clean cage warmed by an infrared heating lamp allowing the animals to regain consciousness. The mice were followed closely post-surgery.

Mice were bled from the tail vein by making a small incision with a blade and blood was collected in a Microvette^®^ CB 300 capillary K2-EDTA tube. Mice were fully conscious during blood sampling, no anesthesia was used. The animals were restrained using a suitable restrainer. Blood samples were immediately placed on melting ice and plasma was obtained by centrifugation at 3,000 rpm for 10 minutes at 4 °C. Isolated plasma was shielded from daylight and stored at -20 °C prior to analysis. Blood samples were taken from 0 to 12 weeks after the first immunization. In total, eight blood samples were obtained from each mouse. Twelve weeks following vaccination mice were sacrificed by CO_2_ inhalation and checked on residual core-shell implants. At the end of the experiment, all samples were tested by indirect ELISA to determine the OVA-specific IgG1 antibody response of the animals.

### Indirect ELISA for OVA-specific IgG1 antibody titers

Flat-bottomed 96-well Nunc-Immuno plates with MaxiSorp surface were coated overnight at room temperature (RT) with 50 μL of a 10 μg/mL OVA in PBS solution. After overnight incubation, the antigen-coated plates were blocked with 100 μL PBS containing 1% (w/v) BSA for 1 h at 37 °C. Plates were then washed three times with wash buffer (0.05% (v/v) Tween 20 in PBS). Three-fold serial dilutions of the plasma samples were prepared in blocking buffer starting at 1:100 and 50 μL of each dilution was added per well. Plates were sealed and incubated at 37 °C for 1 h. Plasma from a naive mouse was included as negative control. Wells containing a 1 wt-% solution of BSA in PBS were used to determine assay background. A monoclonal anti-OVA-IgG1 antibody was used as standard control. After incubation, the well contents were discarded and the plates were washed three times with wash buffer. The wells were then incubated with 50 μL of 0.25 μg/mL biotin-conjugated goat anti-mouse IgG1 diluted in PBS containing 1 wt-% BSA for 1 h at 37 °C. Plates were washed again three times and 50 μL/well of 1:1000 dilution of streptavidin-HRP conjugate was added to the plates and incubated for 30 min at RT. Thereafter, the plates were washed three times with wash buffer and developed with 100 μL/well of the peroxidase substrate reagent, 3,3’,5,5’-TMB, which was incubated for 5 min at RT shielded from light. The colorimetric reaction was stopped by adding 100 μL sulfuric acid 2 N to each well. Absorbance was measured at λ = 450 nm using a Tecan Infinite M1000 microplate reader (Männedorf, Switzerland).

Samples with readings higher than the cut-off value were considered positive. The cut-off value was determined as the absorbance where the mean negative plasma dilution intersects with the mean plate background plus three times the standard deviation of the plate background. The reciprocal of the highest dilution of a plasma sample that has a reading above the cut-off was used as the endpoint titer. IgG1 antibody titers were expressed as log2 value of the highest reciprocal dilution that yielded an OD value above the determined cut-off value.

### Statistics and data analysis

Nonlinear regression analysis was performed to determine the half maximal effective time (ET50). Significance between the ET50 values of the core-shell implants with different polymers as shell material was determined with an extra sum-of-squares F test (alpha = 0.05). Significance between group E (OVA-PBS liquid injection (prime-boost)) and group H (Placebo-PLGA 50:50 core-shell implant + OVA-PBS) after 3 weeks of the OVA-PBS injection was determined with a Mann-Whitney test (alpha = 0.05). This was done to determine whether PLGA had an adjuvant effect. Significance between group A (OVA-PLGA 50:50) and group B (OVA-PLGA 100:0) of the *in vivo* study was determined using an unpaired t test followed by the Holm-Sidak method to correct for multiple comparisons (alpha = 0.05). GraphPad Prism 6.0c was used for all these analyses.

## Results and discussion

### *In vitro* release study: The delayed release of OVA and the influence of lactic to glycolic acid ratio on lag time

**Fig 2:**
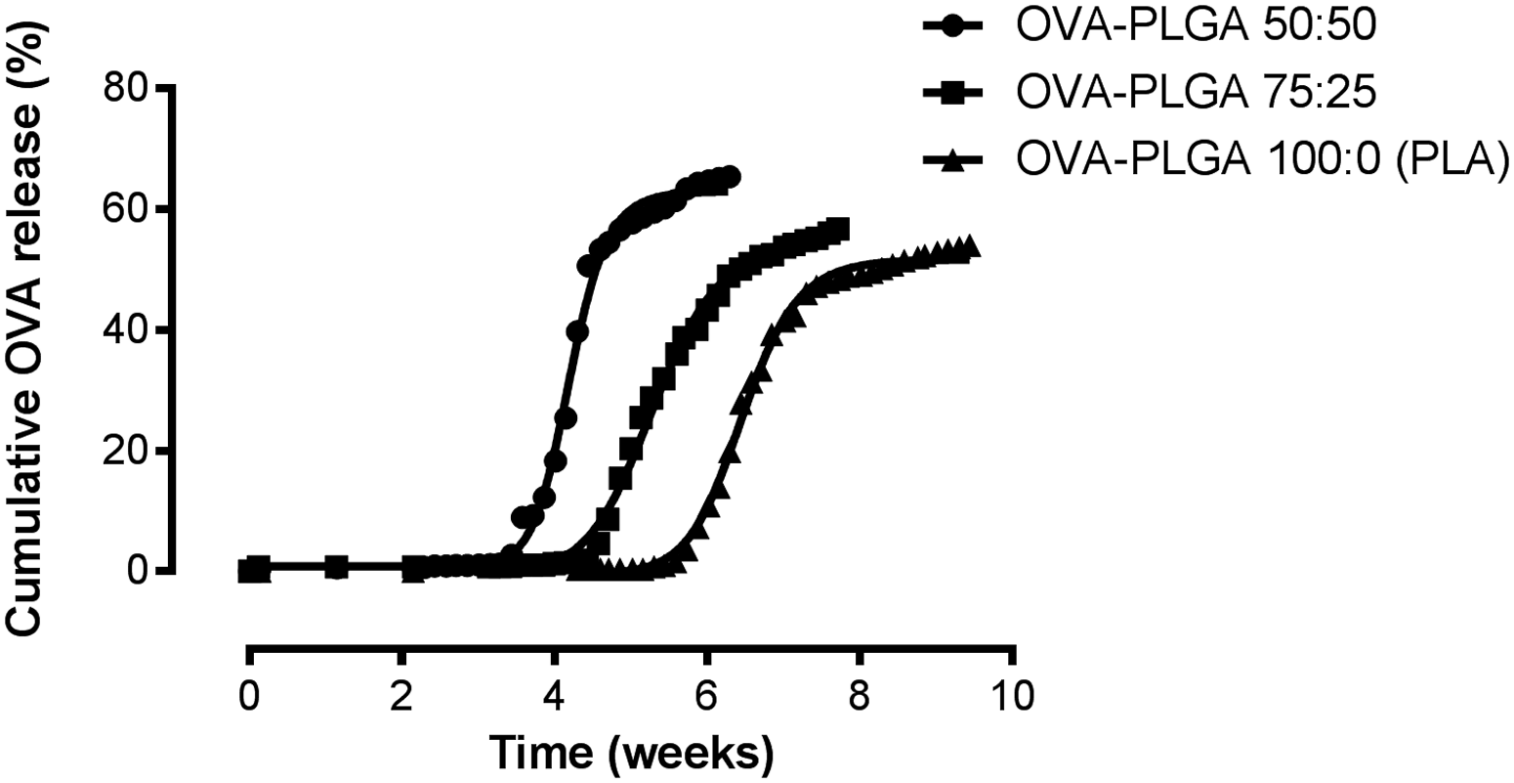
Nonlinear regression analysis of the release of OVA from core-shell implants using PLGA with different lactic to glycolic acid ratios to function as a barrier layer. Calculated ET50 values were 4.2 ± 0.03 weeks, 5.3 ± 0.03 weeks, and 6.5 ± 0.05 weeks for OVA-PLGA 50:50, OVA-PLGA 75:25, and OVA-PLGA 100:0 (PLA), respectively. The difference in ET50 value was statistically significant (P < 0.0001). Results are shown as mean (n = 6).

Fig 2 shows the delayed release of OVA for several shell compositions of the core-shell implants. A clear relation can be observed between the increase of glycolic acid content of the polymer and decrease of lag time. The calculated ET50 values were 4.2 ± 0.03 weeks (R square = 0.92), 5.3 ± 0.03 weeks (R square = 0.97), and 6.5 ± 0.05 weeks (R square = 0.85) for OVA-PLGA 50:50, OVA-PLGA 75:25, and OVA-PLGA 100:0 (PLA), respectively. Comparison of the ET50 values using an extra sum-of-squares F test proved the difference in ET50 values of the different polymers to be statistically significant (P < 0.0001).

These results are in line with PLGA degradation studies conducted by others [8,9]. These studies have shown that an increase of glycolic acid content results in a more hydrophilic and thus a faster degrading polymer. By utilizing this composition dependent degradation profile of PLGA, the lag time, and consequently the delayed release of the active compound, can be tailored by using different lactic to glycolic acid ratios. While other studies that investigated a core-shell implant have shown that the lag time can be influenced by molecular weight of the polymer [10], we showed that varying the lactic to glycolic acid ratio also significantly influences lag time. Fig 2 also shows that the release of OVA is still ongoing after a clear increase. This might indicate a second phase of release at which OVA is slowly diffusing through the PL(G)A shell. Because the focus of this study was to obtain the release of OVA after a certain lag time, sampling was limited to a few weeks after the delayed release.

### Fluorescence spectroscopy and OVA conformation

The use of high temperatures during manufacturing of the implants may denature the antigen. However, in this study the implants were exposed to a temperature of 48 °C for a relatively short time (20 minutes), other studies showed that several proteins, such as DNAse [13], alkaline phosphatase [14], infliximab [15], hepatitis B surface antigen [11], and acyl-homoserine-lactone acylase [16] remained fully stable for several weeks at elevated temperatures (40–60 °C) when incorporated in inulin matrices. Hence, in this study the antigen was freeze-dried with inulin to stabilize the antigen.

**Fig 3:**
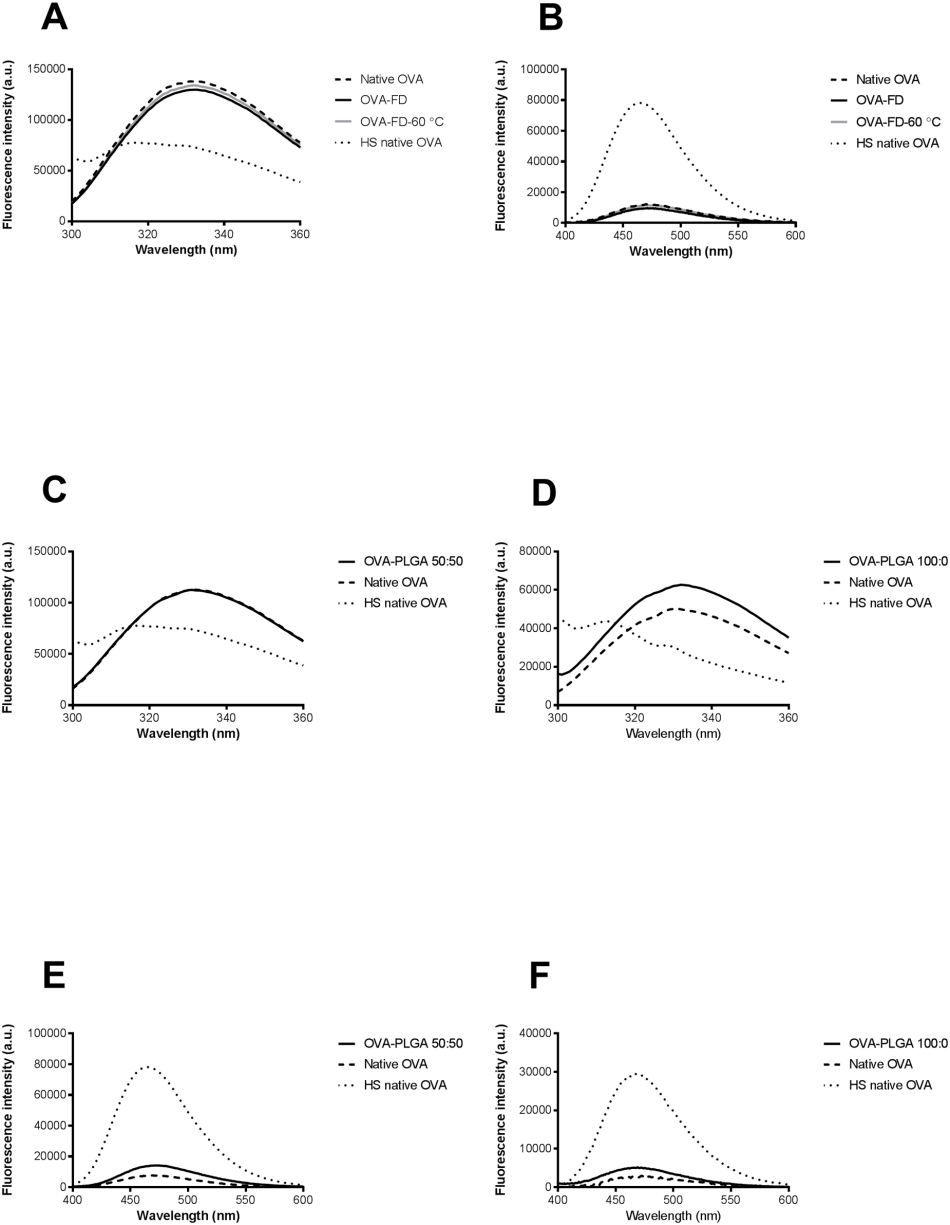
A-F. Intrinsic fluorescence (A) and extrinsic fluorescence (B) of OVA directly after freeze-drying and storage for one week at 60 °C. Intrinsic fluorescence of OVA-PLGA 50:50 (C), OVA-PLGA 100:0 (D), extrinsic fluorescence of OVA-PLGA 50:50 (E), and OVA-PLGA 100:0 (F). Native OVA is shown as a dashed line, while HS-OVA is shown as a dotted line.

Intrinsic and extrinsic fluorescence spectroscopy was performed to investigate whether OVA maintained its native conformation directly after freeze-drying and subsequent storage for one week at 60 °C as freeze-dried powder. In addition, the conformation of OVA within the core was investigated during the lag time. Fig 3A shows the intrinsic fluorescence of native OVA in comparison to HS-OVA, OVA-FD and OVA-FD-60 °C. HS-OVA clearly showed a blueshift and a decrease in fluorescence intensity compared to native OVA. Generally, protein aggregation leads to a blueshift and a change in fluorescence intensity in the intrinsic fluorescence spectra [17]. It can therefore be concluded that heating an aqueous OVA solution for 30 minutes at 100 °C resulted in the aggregation of OVA. The results also show that there is no relevant difference in fluorescence intensity between native OVA and OVA-FD or OVA-FD-60 °C. In addition, no blueshift was observed between these samples. To further investigate a potential conformational change, extrinsic fluorescence spectroscopy was applied using ANS. In aqueous medium, ANS is practically nonfluorescent, hence a low fluorescence intensity is expected for native OVA. However, protein aggregation leads to a significant increase in fluorescence intensity and a blueshift since ANS interacts with hydrophobic pockets that may be present on protein aggregates [17]. Indeed, a clear increase in fluorescence intensity and a blueshift can be observed for HS-OVA compared to native OVA (Fig 3B). Furthermore, Fig 3B also shows that there is no relevant difference in fluorescence intensity between native OVA and the freeze-dried OVA samples. Moreover, OVA-FD and OVA-FD-60 °C did not show any blueshift compared to native OVA. Fig 3C and 3D show the intrinsic fluorescence of native OVA in comparison to HS-OVA, OVA-PLGA 50:50 and OVA-PLGA 100:0. The intrinsic fluorescence spectra of OVA-PLGA 50:50 and OVA-PLGA 100:0 did not show a blueshift. However, OVA-PLGA 100:0 did show an increase in overall fluorescence intensity. This might indicate that tryptophan groups are more prominently excited, suggesting a possible conformational change. However, the extrinsic fluorescence spectra of OVA-PLGA 50:50 and OVA-PLGA 100:0 showed only a slight increase in fluorescence intensity, a clear blueshift compared to native OVA was not visible (Fig 3E and 3F).

The results indicate that freeze-drying OVA in the presence of inulin, and subsequent storage of freeze-dried OVA for one week at 60 °C, does not affect the conformation of OVA. Furthermore, minimal differences in OVA conformation between native OVA and OVA from core-shell implants that were incubated until just before the *in vitro* release was observed. However, these results do not indicate that the conformational change of other proteins would show a similar outcome. Therefore, protein stability should be investigated case by case.

### Systemic IgG1 antibody titers induced by subcutaneous implantation of OVA-containing core-shell implants

Based on the *in vitro* results an *in vivo* experiment in mice was conducted. Core-shell implants containing the model antigen OVA were surgically inserted s.c. to investigate whether systemic OVA-specific IgG1 antibody titers were induced after a certain lag time. PLGA 50:50 and PLGA 100:0 were used to tailor the lag time of OVA release from the core-shell implants. For unknown reasons, two mice of groups A (OVA-PLGA 50:50 core-shell implant) and F (Placebo-PLGA 50:50 core-shell implant), and 1 mouse of groups B (OVA-PLGA 100:0 core-shell implant) and G (Placebo-PLGA 100:0 core-shell implant), deceased shortly after the surgery.

**Fig 4:**
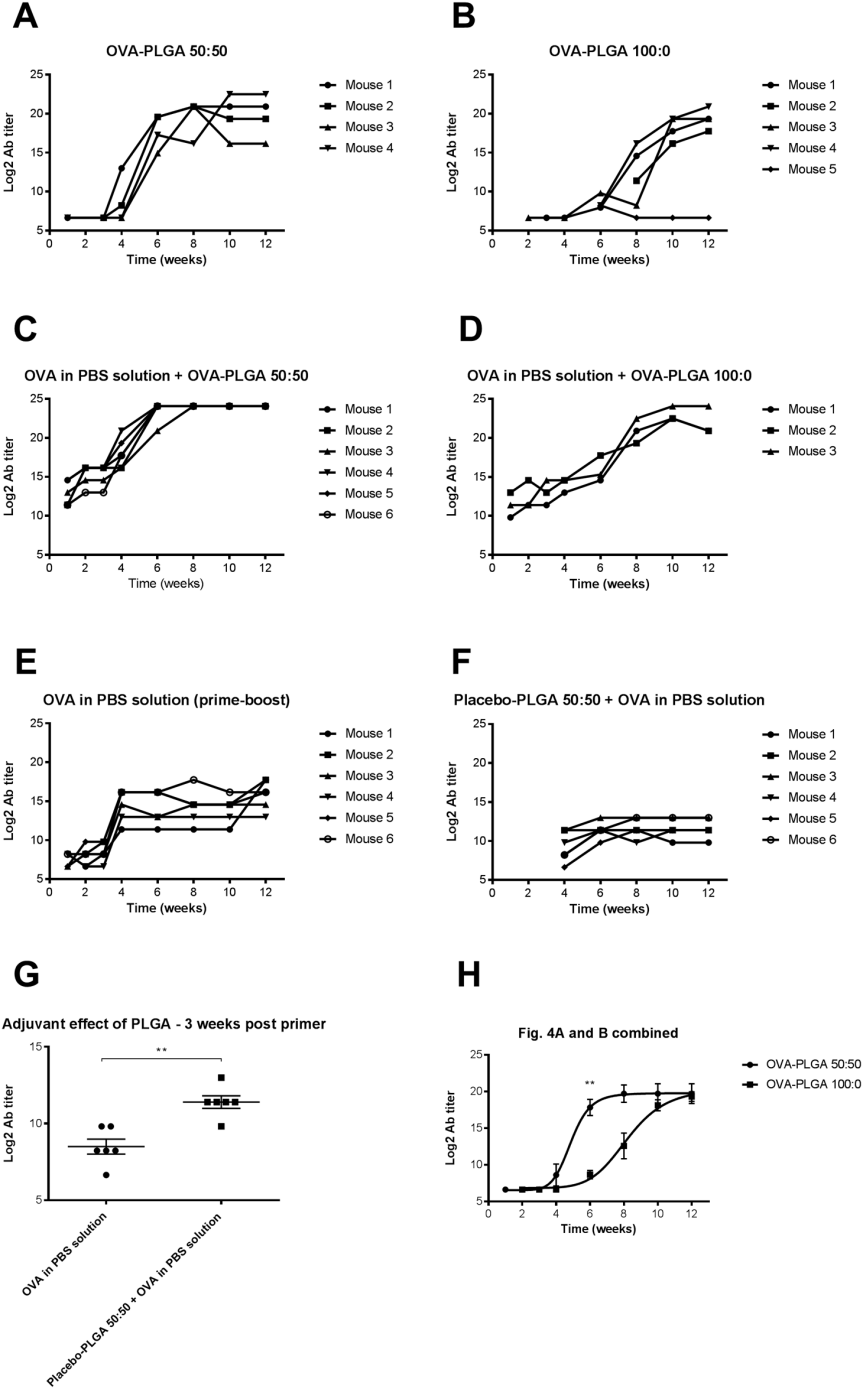
A-H. IgG1 antibody titers over time after immunization. Mice were immunized with OVA-PLGA 50:50 core-shell implant (A), with OVA-PLGA 100:0 core-shell implant (B), with OVA dissolved in PBS together with OVA-PLGA 50:50 core-shell implant (C), with OVA dissolved in PBS together with OVA-PLGA 100:0 core-shell implant (D), with OVA dissolved in PBS, primer (day 0) and booster (day 21) (E), placebo-PLGA50:50 core-shell implant (day 0) followed by OVA dissolved in PBS at day 21 (F). OVA in PBS solution (prime-boost) vs. placebo-PLGA 50:50 + OVA in PBS solution 3 weeks after injection of the primer to investigate adjuvant effect of PLGA. Results are shown as mean ± SEM. ** = P ≤ 0.01 (G). Nonlinear regression analysis of OVA-PLGA 50:50 and OVA-PLGA 100:0 data (poor responder mouse 5 not included) was performed with GraphPad Prism 6.0c. Calculated ET50 for OVA-PLGA 50:50 = 4.9 ± 0.4 weeks and OVA-PLGA 100:0 = 8.2 ± 0.5 weeks. Results are shown as mean ± SEM. ** = P ≤ 0.01 (H). Plasma was collected at regular time points after vaccination. The antibody levels measured in each individual mouse during the study are presented in the graph. For some time points there are no data points depicted in the graph because no antibodies were detected at that time point (mouse 2, Fig 4B).

To ensure that core-shell implants without OVA did not induce any unwanted IgG1 antibody response, placebo cores were used in combination with PLGA 50:50 (group F) and PLGA 100:0 (group G) as barrier. As expected, the placebo groups did not show any antibody response. That is, the plasma samples of the placebo groups did not have an OD reading above the determined cut-off. The cut-off was determined based on plasma taken from a healthy non-vaccinated mouse (mean absorbance ± SD at 1:100 dilution = 0.156 ± 0.059) and the plate background determined with 1% BSA-PBS (mean absorbance ± SD at 1:100 dilution = 0.094 ± 0.034). Fig 4A shows the OVA-specific IgG1 antibody response after the s.c. insertion of an OVA-containing core-shell implant using PLGA 50:50 as barrier (group A). No antibody titers were observed for 3 weeks. Interestingly, after 4 weeks a clear increase in antibody response was measured. Since it takes approximately one week to detect OVA-specific antibody titers [18,19], these data indicate that the OVA was released after about 3 weeks from the core. These results are in line with the *in vitro* release data (Fig 2), where OVA was released after about 3 weeks.

The same trend can be observed for the antibody response measured in the group of mice that were immunized with the OVA-containing core-shell implant, using PLGA 100:0 as barrier (group B). Fig 4B shows that the IgG1 antibody titers clearly increased between week 6 and 8. These results are also in line with the *in vitro* data (Fig 2), which showed a release of OVA after 5–6 weeks. In addition, at week 6, the data in Fig 4H shows a significant difference in IgG1 antibody titers between both formulations (P ≤ 0.01). After performing a nonlinear regression analysis on both groups, the calculated ET50 for OVA-PLGA 50:50 and OVA-PLGA 100:0 were 4.9 ± 0.4 weeks (R square = 0.88) and 8.2 ± 0.5 weeks (R square = 0.90), respectively. In one mouse very low antibody titers were detected, possibly because this mouse was a poor responder, or due to incomplete release of OVA from the core-shell implant. Therefore, the antibody titers generated by this mouse were not used in the nonlinear regression analysis. However, as in the case of the *in vitro* data, a clear trend can be observed in the lag time between the core-shell formulations containing PLGA 50:50 and PLGA 100:0 as barrier.

After priming the mice s.c. with OVA in PBS solution and surgical insertion of an OVA-PLGA 50:50 or OVA-PLGA 100:0 core-shell implant at the same time (groups C and D, respectively), the antibody titers were elevated after one week due to the prime vaccination. Interestingly, 4 weeks after priming a clear increase of the plasma IgG1 titers was measured for the OVA-PLGA 50:50 core-shell implant (Fig 4C). In addition, 6 weeks after the priming a clear increase of the plasma IgG1 titers was also observed for the OVA-PLGA 100:0 core-shell implant (Fig 4D). The increase of plasma IgG1 titers is most likely a result of the OVA release from the core-shell implants presumably between week 3 and week 6 and between week 5 and 7 for the OVA-PLGA 50:50 and OVA-PLGA 100:0 core-shell implant, respectively. Furthermore, mice that were s.c. primed and boosted with a solution of OVA in PBS (group E) had elevated IgG1 antibody titers from week 1 onwards, meaning that the conventional prime-boost vaccination was effective (Fig 4E). However, from week 1 onwards the level of IgG1 titers produced after conventional prime-boost vaccination was significantly lower than the IgG1 titers observed using the core-shell configuration (Fig 4C and 4D). This led to the assumption that PLGA asserted a potential adjuvant effect, since PLGA is known to have adjuvant properties [20]. To investigate the potential adjuvant effect of PLGA, mice were immunized with OVA in PBS solution 3 weeks (day 21) after the insertion of a placebo-PLGA 50:50 core-shell implant (group H). Indeed, the plasma IgG1 titers are clearly increased at week 4 followed by a plateau from week 6 onwards and are significantly higher in comparison with the conventional prime-boost group 3 weeks post primer (Fig 4F and 4G). That is, delayed release of OVA caused higher IgG1 antibody titers.

The results described in this study clearly indicate that it is possible to achieve an adjustable delayed IgG1 specific antibody response using OVA as model antigen in a core-shell implant with PL(G)A. The results of our study are in line with a recently published study from Tzeng et al. The study from Tzeng et al. showed two bursts of inactivated polio vaccine one month apart in a microsphere containing, single administration formulation [21]. Although the production method of the core-shell formulation described in the present study has advantages over the production of microspheres (e.g. absence of organic solvents, and induction of mechanical stresses such as vortexing, centrifugation or sonication), further research is required to improve its design. Obviously, administration of a vaccine by the procedure used in this study, i.e. s.c. insertion of the implant by surgical intervention, is clinically not acceptable. Recently, McHugh et al. described a promising fabrication method, termed StampEd Assembly of polymer Layers (SEAL), to create injectable pulsatile drug-delivery microparticles that might be suitable as a single-injection vaccine [22]. In general, possible improvements would be to produce the core-shell implant as a rod-shaped device that fits in a needle, which makes administration by s.c. or intramuscular injection possible. Furthermore, the core-shell implant only included the booster but not the primer. Including the primer might be possible by providing the rod-shaped device with a fast dissolving coating containing the antigen. Moreover, even a primer with two boosters could be possible; the primer is released from a fast dissolving coating, followed by the release of a first booster and a second booster after the degradation of suitable polymers providing for a short and long lag time, respectively. Production techniques such as hot-melt co-extrusion might help to improve the design of the core-shell by extruding several layers of material into small rod-shaped implants.

The findings presented in our study might further facilitate the development of a single-injection formulation that provides for a prime-boost immunization.

## Conclusions

The main goal of this study was to investigate the feasibility of a single-administration vaccine by using a core-shell based implant, using PL(G)A as a barrier, to induce a certain lag time before antigen release. This study has shown that the ratio of lactic to glycolic acid plays an important role in the lag time at which the antigen is released; an increase in glycolic acid content results in a decrease in lag time. In addition, OVA within the core did not seem to be affected during the lag time before release. Furthermore, OVA encapsulated in the core-shell implant induced a delayed OVA-specific IgG1 antibody response in mice higher than that elicited by conventional s.c. vaccination with OVA in PBS solution. These findings will contribute to the further development of a single-administration vaccine.
